# Different Appearance of Chest CT Images of T2DM and NDM Patients with COVID-19 Pneumonia Based on an Artificial Intelligent Quantitative Method

**DOI:** 10.1155/2021/6616069

**Published:** 2021-03-11

**Authors:** Shan Lu, Zhiheng Xing, Shiyu Zhao, Xianglu Meng, Juhong Yang, Wenlong Ding, Jigang Wang, Chencui Huang, Jingxu Xu, Baocheng Chang, Jun Shen

**Affiliations:** ^1^NHC Key Laboratory of Hormones and Development (Tianjin Medical University), Tianjin Key Laboratory of Metabolic Diseases, Tianjin Medical University Chu Hsien-I Memorial Hospital and Tianjin Institute of Endocrinology, Tianjin, China; ^2^Haihe Hospital, Tianjin University, Tianjin Institute of Respiratory Diseases, Tianjin, China; ^3^Department of Research Collaboration, R&D Center, Beijing Deepwise & League of PHD Technology Co. Ltd, Beijing, China

## Abstract

COVID-19 is a kind of pneumonia with new coronavirus infection, and the risk of death in COVID-19 patients with diabetes is four times higher than that in healthy people. It is unclear whether there is a difference in chest CT images between type 2 diabetes mellitus (T2DM) and non-diabetes mellitus (NDM) COVID-19 patients. The aim of this study was to investigate the differences in chest CT images between T2DM and NDM patients with COVID-19 based on a quantitative method of artificial intelligence. A total of 62 patients with COVID-19 pneumonia were retrospectively enrolled and divided into group A (T2DM COVID-19 pneumonia group, *n* = 15) and group B (NDM COVID-19 pneumonia group, *n* = 47). The clinical and laboratory examination information of the two groups was collected. Quantitative features (volume of consolidation shadows and ground glass shadows, proportion of consolidation shadow (or ground glass shadow) to lobe volume, total volume, total proportion, and number) of chest spiral CT images were extracted using Dr. Wise @Pneumonia software. The results showed that among the 26 CT image features, the total volume and proportion of bilateral pulmonary consolidation shadow in group A were larger than those in group B (*P*=0.031 and 0.019, respectively); there was no significant difference in the total volume and proportion of bilateral pulmonary ground glass density shadow between the two groups (*P* > 0.05). In group A, the blood glucose level was correlated with the volume of consolidation shadow and the proportion of consolidation shadow to right middle lobe volume, and higher than those patients in group B. In conclusion, the inflammatory exudation in the lung of COVID-19 patients with diabetes is more serious than that of patients without diabetes based on the quantitative method of artificial intelligence. Moreover, the blood glucose level is positively correlated with pulmonary inflammatory exudation in COVID-19 patients.

## 1. Introduction

COVID-19 is a rapidly spreading disease that has reached the pandemic scale, affecting more than 100 countries worldwide. To date, the epidemic has swept over more than 200 countries and regions. The number of new coronavirus infections in the world has exceeded 6.81 million, and the cumulative number of deaths has exceeded 0.396 million. On January 30, 2020, the WHO (World Health Organization) declared the epidemic a public health emergency of international concern. On March 11, 2020, the WHO characterized the epidemic as a pandemic disease.

The characteristic of COVID-19 pneumonia is feathered with aggregation, which is more likely to affect elderly men with underlying diseases and cause serious or even fatal respiratory diseases [1, 2]. It has been reported that diabetes mellitus (DM) is a major age-independent risk factor for the severity of COVID-19 pneumonia [3]. The mortality risk of novel coronavirus pneumonia in DM patients is 4 times that of healthy people [4]. Whether there is a difference in chest CT images between type 2 diabetes mellitus (T2DM) and nondiabetes mellitus (NDM) COVID-19 patients is still unknown. In this study, an artificial intelligence quantitative method was used to evaluate the differences in chest CT images between T2DM and NDM patients with COVID-19 pneumonia.

## 2. Materials and Methods

Patients with COVID-19 pneumonia in Tianjin Haihe Hospital from February 2020 to April 2020 were retrospectively enrolled, and according to whether the novel coronavirus pneumonia patients were T2DM, they were divided into group A (COVID-19 T2DM group) and group B (COVID-19 NDM group). Clinical and laboratory examination information of the two groups was collected.

**Figure 1:**
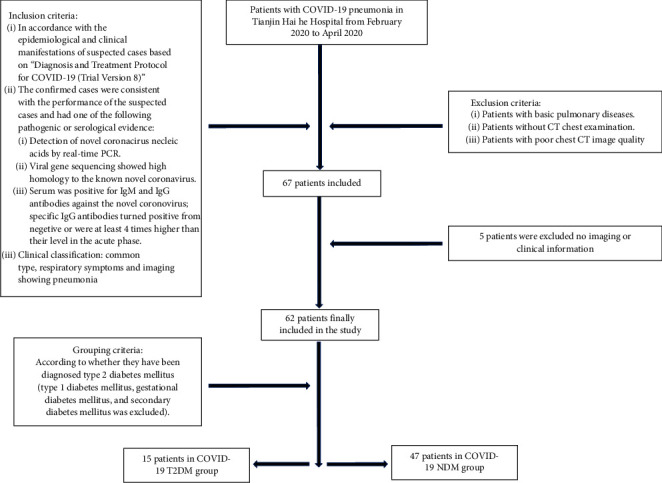
The inclusion and exclusion criteria of the study.

The inclusion and exclusion criteria are as provided in Figure 1.

### 2.1. Inspection Equipment and Methods

A spiral CT scanner (Aquilion Prime 128, Canon Medical Systems, Otawara, Japan) present in Tianjin Haihe Hospital was used to perform the thin slice CT scan from the lung apex to the adrenal gland during a full inspiration. CT scanning parameters were as follows: tube voltage 120 kV, tube current 500 mAs, layer spacing 0.8 mm, and layer thickness 1 mm. A hybrid iterative reconstruction algorithm (AIDR 3D) was used. Window level was 400 HU, and window width was 40 HU.

### 2.2. Data Feature Extraction

**Figure 2:**
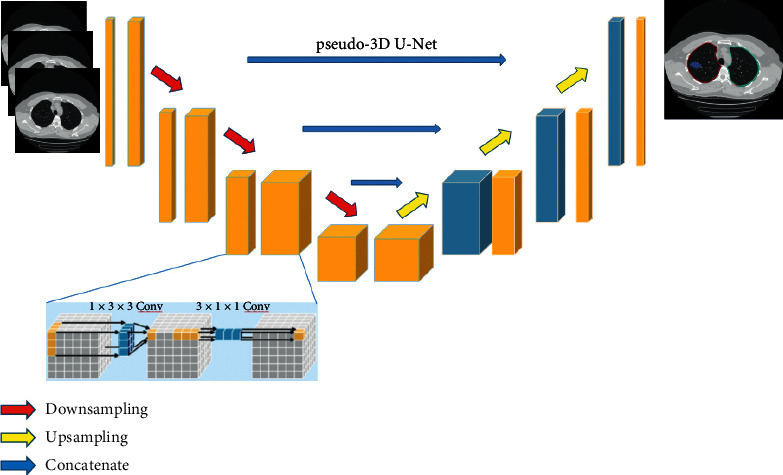
Lung infection and labeling and volume calculation were used to determine the volume of pulmonary inflammation.

The CT features were extracted using Dr. Wise @Pneumonia software (Beijing Deepwise & League of PhD Technology Co. Ltd., China). Lung lobe segmentation, lung infection and labeling, and volume calculation were performed by a commercially available deep learning algorithm developed for pulmonary pneumonia. The segmentation network was based on the U-Net architecture, which employs pseudo-3D convolution as its basic blocks. Multiple CT slices were used to form the 3D input. The established U-Net was used as the building architecture for infection segmentation. To efficiently exploit the 3D context information for autocontouring of possible infection regions, in our study, the 3D convolutional layers were replaced by two 2D convolutional layers. Using such pseudo-3D convolutional layers, the proposed model acquired the ability of 3D context modeling, as shown in Figure 2. The extracted image feature parameters included the volume of the ground glass shadow and consolidation shadow, the proportion of the lung lobe, the total volume, the total proportion, and the number.

### 2.3. Statistical Analysis

Statistical software (R 3.6.2) was used. The measurement data were normally distributed and are expressed as mean ± standard deviation ($\bar{x}\pm s$). Count data are expressed as frequencies and percentages (%). For the comparison of the clinical and imaging indexes between group A and group B, the chi-square test was used for counting data, the *t*-test was used for measuring data and counting data, and multiple linear regression analysis was used to analyze the correlation of imaging indexes with clinical features and blood glucose in group A. *P* < 0.05 was defined as statistically significant.

## 3. Results

### 3.1. Composition of the Clinical Data

In this study, 67 patients (36 men and 31 women) with COVID-19 pneumonia were included, including 16 patients with T2DM and 51 with NDM. One patient with T2DM COVID-19 pneumonia and 4 patients with NDM COVID-19 pneumonia had no imaging or clinical information and were excluded. There were 15 patients in group A (42–78 years old, 61 ± 11 years old), including 9 men and 6 women, and 47 patients in group B (43–89 years old, 60 ± 12 years old), including 18 men and 29 women. There was no significant difference in age or sex between the two groups.

### 3.2. Clinical Data and Laboratory Examination Indexes between Group A and Group B

The results showed no significant difference in sex, age, fever status, admission temperature, routine blood tests, CK-MB, etc. (*P* > 0.05), as shown in Table 1.

### 3.3. Image Feature Extraction

**Figure 3:**
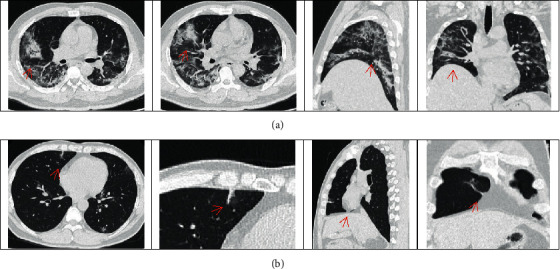
Chest CT findings in COVID-19 patients with T2DM and NDM. (a) Male, 49 years old, common type COVID-19, 10-year history of T2DM. Patchy shadows can be seen in multiple lobes of both lungs, local consolidation can be seen in the middle lobe of the right lung, and air bronchi signs can be seen inside. (b) Male, 41 years old, common type COVID-19, NDM. Patchy shadows are seen in multiple lobes of both lungs, with local consolidation in the middle lobe of the right lung.

In this study, 26 imaging features were extracted. There were significant differences in the total volume and proportion of bilateral lung consolidation between group A and group B (*P*=0.031 and 0.019, respectively), and the total volume and total proportion of the bilateral consolidation in group A were larger than those in group B, as shown in Figure 3. There was no significant difference in the imaging indexes of the ground glass opacity between the two groups (*P* > 0.05), as shown in Table 2.

### 3.4. Correlation of Imaging Indexes with Clinical Indexes and Blood Glucose in Group A

The results showed that blood glucose was a significant factor affecting the total volume and proportion of consolidation shadow. The total volume of consolidation shadow was significantly correlated with blood glucose, neutrophils, heartache, and CRP. The proportion of consolidation shadow was significantly correlated with blood glucose, headache, IDH, and fever. The total volume and proportion of consolidation shadow in patients with DM were 262.12 (*P*=0.011) and 13.63 (*P* < 0.001) higher than those in patients without DM, as shown in Table 3.

## 4. Discussion

COVID-19 is a coated beta RNA virus that is widely found in mammals and birds. It belongs to the same subgenus as SARS and some bat coronaviruses. Genome sequencing analysis showed that the similarity between COVID-19 and SARS virus sequences was 79% [5–8]. COVID-19 and SARS virus both bind to the angiotensin converting enzyme 2 (ACE2) receptor of host cells through the receptor binding domain on the surface of the key component S protein; however, the affinity of COVID-19 surface protein to ACE2 of host cells is 10 to 20 times higher than that of the SARS virus [9]. COVID-19 enters human cells by binding to the ACE2 receptor in the respiratory tract and lung tissue, which triggers a series of respiratory reactions.

After COVID-19 infection, the main pathological manifestations of both lungs are thickening of the interlobular septum and exudative changes in the alveoli, which are reflected on the chest CT that showed multiple ground glass densities or fine mesh shadows distributed under the pleura of one or both lungs. When the thickening of the intralobular septum was similar to that of the alveolar exudation, it was mainly manifested as ground glass opacity, which could be nodular, patchy, or even largely lobular; when the thickening degree of the intralobular septum was more severe than that of the alveolar exudation, it showed multiple fine grid shadows in ground glass density shadows, showing gravel road signs. The imaging findings of novel coronavirus pneumonia include halo sign, anti-halo sign, vascular thickening sign, and bronchiectasis sign. When the alveolar exudation is serious, the ground glass density shadows can be solid shadows. When there is an obvious large consolidation shadow, we should be highly alert to the possible rapid deterioration of the patient.

DM patients have reduced immune cells, decreased NKT cell activity, abnormal immune function, and low immune responsiveness. Its complications may involve multiple organs of the body. Therefore, DM patients are a high-risk population for various acute or chronic infections [10]. Huang et al. retrospectively analyzed 41 cases of patients with coronavirus infection in Wuhan, among whom 32% of the patients had complications, 20% were complicated with DM, and 15% were complicated with hypertension, suggesting that DM patients are likely to be extra susceptible to coronavirus [11]. Some studies have shown that the proportion of DM in COVID-19 patients is 10.1%–20.0% and that of critically ill patients is 22.2% [2, 11, 12]. Further investigation suggested that the severe rate of COVID-19 patients with DM may be higher, which is consistent with the research results of Zhang JJ, who found that the severe rate and mortality rate of COVID-19 patients with diabetes mellitus are higher than those without complications [13].

Data from several clinical retrospective studies were analyzed, and it was found that hyperglycemia and diabetes had similar gene structures and they were independent risk factors for death after SARS infection [14–16]. Our results showed that there was no difference in the volume or total volume of the ground glass density shadows in each lung lobe between the two groups; however, the total volume of consolidation and total volume of the consolidation/total volume of the lung lobe in the COVID-19 diabetes group were higher than those in the COVID-19 NDM group (*P* < 0.05). These results indicated that novel coronavirus pneumonia patients with DM are in a more inflammatory state than those with NDM; that is, their pneumonia is more serious than in those with NDM, and their severity of lung involvement on CT is related to the severity of their disease. These results further suggest that novel coronavirus pneumonia patients with DM have a higher incidence of severe disease, which is consistent with the above findings. It may be related to immune disorders and metabolic disorders in DM patients, leading to decreased ability of the body to clear the virus and rapid disease progression after infection with COVID-19.

Previous studies have found that ACE2 is also expressed in pancreatic endocrine tissues in addition to the respiratory tract and lung tissues. The SARS virus may invade and destroy pancreatic islet cells by binding to this functional receptor. Therefore, COVID-19, which is similar to SARS and has the same receptor, may also invade pancreatic islet tissue by combining with the receptor, directly or indirectly damaging islet *β* cells to accelerate the progression of DM [17]. COVID-19 infection can also increase the burden on organs, immune dysfunction, inflammatory storms, and endocrine imbalance to further promote the development of diabetes and the interactions between them to form a vicious cycle. In this study, the blood glucose level was positively correlated with the volume of consolidation in the middle lobe of the right lung and the volume of consolidation in the middle lobe of the right lung/volume of the middle lobe of the right lung. These results cannot explain why blood glucose levels are related to the severity of pneumonia, and a more rigorous experimental design and a larger sample size are needed for further exploration.

In conclusion, this study suggested that patients with COVID-19 pneumonia complicated with diabetes had higher pulmonary inflammatory exudation than those without diabetes; that is, patients with COVID-19 pneumonia complicated with diabetes may have a higher rate of severe disease, suggesting that clinicians should pay more attention to the treatment of patients with COVID-19 pneumonia complicated with diabetes. This study further confirms that blood glucose levels are positively correlated with pulmonary inflammatory exudation in patients with COVID-19 pneumonia but does not indicate that blood glucose levels are correlated with the severity of COVID-19 pneumonia. There are still some limitations of this study. The number of patients with COVID-19 combined with diabetes is relatively small, and the conclusions drawn need to be further explored and verified by a more rigorous experimental design and a larger sample size.

## Figures and Tables

**Table 1:** Comparison of clinical characteristics between group A and group B.

	Group A	Group B	*P*
Sex (male/female)	9/6	18/29	0.146
Age (year)	61 ± 11	60 ± 12	0.813
Fever (cases)	93.3% (14/15)	80.9% (38/47)	0.252
Dry cough (cases)	66.7% (10/15)	44.7% (21/47)	0.138
Nausea (cases)	6.7% (1/15)	6.4% (3/47)	0.969
Headache (cases)	0% (0/15)	4.3% (2/47)	0.417
Diarrhea (cases)	13.3% (2/15)	12.8% (6/47)	0.954
Admission temperature (°C)	37.2 ± 1.2	36.9 ± 0.7	0.401
Admission heart rate (beats/min)	85.5 ± 13.9	84.8 ± 16.6	0.967
White blood cell count (×10^9^/L)	5.3 ± 2.8	5.4 ± 2.4	0.981
Neutrophil count (×10^9^/L)	3.8 ± 2.7	3.6 ± 2.1	0.745
Lymphocyte count (×10^9^/L)	1.0 ± 0.5	1.2 ± 0.5	0.189
CRP (mg/L)	37.6 ± 27.5	22.4 ± 35.3	0.100
D-Dimer (mg/L)	0.8 ± 0.8	0.6 ± 0.4	0.177
CK (U/L)	89.4 ± 95.2	134.5 ± 284.5	0.109
LDH (U/L)	556.3 ± 198.0	547.1 ± 182.3	0.338
Monocyte count (×10^9^/L)	0.5 ± 0.3	0.6 ± 0.4	0.433
Platelet (×10^9^/L)	175.2 ± 83.5	191.1 ± 51.8	0.621
PCT (ng/ml)	0.05 ± 0.03	0.05 ± 0.03	0.827
CK-MB (U/L)	8.1 ± 3.4	11.1 ± 15.8	0.234

**Table 2:** Comparison of imaging features between group A and group B.

Consolidation		Group A	Group B	*P*
Shadow	Upper lobe of right lung_volume	52.3 ± 97.6	12.8 ± 52.7	0.152
	Upper lobe of right lung_proportion of pulmonary lobes	7.7 ± 15.1	1.2 ± 4.6	0.121
	Lower lobe of right lung_volume	299.5 ± 330.1	108.4 ± 259.4	0.054
	Lower lobe of right lung_proportion of pulmonary lobes	27.6 ± 29.9	10.4 ± 20.0	0.051
	Middle lobe of right lung_volume	2.5 ± 8.9	3.6 ± 17.3	0.752
	Middle lobe of right lung_proportion of pulmonary lobes	1.4 ± 5.2	0.5 ± 2.0	0.513
	Upper lobe of left lung_volume	18.3 ± 55.3	25.1 ± 57.2	0.686
	Upper lobe of left lung_proportion of pulmonary lobes	1.7 ± 4.6	3.1 ± 7.4	0.385
	Lower lobe of left lung_volume	183.6 ± 199.5	99.8 ± 212.1	0.176
	Lower lobe of left lung_proportion of pulmonary lobes	20.0 ± 24.2	11.6 ± 21.5	0.236
	Total volume	556.1 ± 447.4	249.7 ± 462.0	0.031
	Total proportion	25.0 ± 18.4	11.3 ± 17.2	0.019
	Number	2.5 ± 18.4	2.0 ± 1.3	0.211
Ground glass	Upper lobe of right lung_volume	8.1 ± 16.6	4.3 ± 17.6	0.452
	Upper lobe of right lung_proportion of pulmonary lobes	0.8 ± 1.5	0.4 ± 1.2	0.299
	Lower lobe of right lung_volume	14.4 ± 29.2	22.7 ± 97.2	0.606
	Lower lobe of right lung_proportion of pulmonary lobes	1.3 ± 2.6	1.5 ± 5.3	0.884
	Middle lobe of right lung_volume	2.2 ± 3.7	3.3 ± 15.0	0.652
	Middle lobe of right lung_proportion of pulmonary lobes	0.4 ± 0.6	0.7 ± 3.0	0.518
	Upper lobe of left lung_volume	37.6 ± 110.5	0.8 ± 1.7	0.219
	Upper lobe of left lung_proportion of pulmonary lobes	2.7 ± 7.4	0.1 ± 0.2	0.190
	Lower lobe of left lung_volume	26.2 ± 58.4	12.5 ± 59.9	0.440
	Lower lobe of left lung_proportion of pulmonary lobes	2.4 ± 5.1	0.9 ± 3.7	0.328
	Total volume	88.4 ± 162.5	43.7 ± 158.4	0.360
	Total proportion	2.2 ± 3.1	1.8 ± 4.2	0.730
	Number	2.3 ± 1.8	1.8 ± 1.4	0.390

**Table 3:** Multiple regression analysis of factors affecting consolidation features^*∗*^.

Imaging features	Factors	Coefficients (*β*)	Std. error	Standardized coefficients	95% CI	*P* value
Total volume	Blood glucose	261.12	99.00	0.24	62.87–459.36	0.011
	Neutrophils	70.69	21.42	0.33	27.80–113.59	0.002
	Headache	1058.267	237.59	0.40	582.50–1534.03	<0.001
Total proportion	CRP	5.03	1.42	0.36	2.18–7.88	0.001
	Blood glucose	13.63	4.45	0.32	4.72–22.55	0.003
	Headache	42.78	10.89	0.42	20.97–64.59	<0.001
	IDH	0.034	0.01	0.34	0.01–0.06	0.002
	Fever	12.29	5.23	0.25	1.82–22.77	0.022

^*∗*^Adjusted personal and clinical characteristics listed in Table 1. *F* = 17.95, *P* < 0.001, adjusted *R* square = 0.350; *F* = 9.206, *P* < 0.001, adjusted *R* square = 0.526.

## Data Availability

The data used to support the findings of this study are available from the corresponding author upon request.
